# Assessment and improvement of HIV screening rates in a Midwest primary care practice using an electronic clinical decision support system: a quality improvement study

**DOI:** 10.1186/s12911-016-0320-5

**Published:** 2016-07-04

**Authors:** Jasmine R. Marcelin, Eugene M. Tan, Alberto Marcelin, Marianne Scheitel, Praveen Ramu, Ronald Hankey, Pritesh Keniya, Majken Wingo, Stacey A. Rizza, Frederick North, Rajeev Chaudhry

**Affiliations:** Division of Infectious Diseases, Mayo Clinic, 200 1st St SW, Rochester, MN 55905 USA; Department of Internal Medicine, Mayo Clinic, 200 1st St SW, Rochester, MN 55905 USA; Department of Family Medicine, Mayo Clinic, 200 1st St SW, Rochester, MN 55905 USA; Department of Information Technology Administration, Mayo Clinic, 200 1st St SW, Rochester, MN 55905 USA

**Keywords:** HIV, Universal screening, Clinical decision support systems, Quality improvement

## Abstract

**Background:**

Universal human immunodeficiency virus (HIV) screening remains low in many clinical practices despite published guidelines recommending screening for all patients between ages 13–65. Electronic clinical decision support tools have improved screening rates for many chronic diseases. We designed a quality improvement project to improve the rate of universal HIV screening of adult patients in a Midwest primary care practice using a clinical decision support tool.

**Methods:**

We conducted this quality improvement project in Rochester, Minnesota from January 1, 2014 to December 31, 2014. Baseline primary care practice HIV screening data were acquired from January 1, 2014 to April 30, 2014. We surveyed providers and educated them about current CDC recommended screening guidelines. We then added an HIV screening alert to an existing electronic clinical decision support tool and post-intervention HIV screening rates were obtained from May 1, 2014 to December 31, 2014. The primary quality outcome being assessed was change in universal HIV screening rates.

**Results:**

Twelve thousand five hundred ninety-six unique patients were eligible for HIV screening in 2014; 327 were screened for HIV. 6,070 and 6,526 patients were seen before and after the intervention, respectively. 1.80 % of eligible patients and 3.34 % of eligible patients were screened prior to and after the intervention, respectively (difference of −1.54 % [−2.1 %, −0.99 %], *p* < 0.0001); OR 1.89 (1.50, 2.38). Prior to the intervention, African Americans were more likely to have been screened for HIV (OR 3.86 (2.22, 6.71; *p* < 0.001) than Whites, but this effect decreased significantly after the intervention (OR 1.90 (1.12, 3.21; *p* = 0.03).

**Conclusions:**

These data showed that an electronic alert almost doubled the rates of universal HIV screening by primary care providers in a Midwestern practice and reduced racial disparities, but there is still substantial room for improvement in universal screening practices. Opportunities for universal HIV screening remain abundant, as many providers either do not understand the importance of screening average risk patients or do not remember to discuss it. Alerts to remind providers of current guidelines and help identify screening opportunities can be helpful.

**Electronic supplementary material:**

The online version of this article (doi:10.1186/s12911-016-0320-5) contains supplementary material, which is available to authorized users.

## Background

### Importance of universal HIV screening

The Centers for Disease Control and Prevention (CDC) estimates that greater than 1.2 million people ≥13 years in the United States are infected with the human immunodeficiency virus (HIV), 14 % of whom are unaware of their HIV infection [1]. While the incidence of new HIV infections has remained relatively stable over the years, almost 50,000 persons were diagnosed with HIV in 2013 [1]. Previous HIV screening guidelines recommended risk-based screening for HIV in adolescents and adults. However, this approach fails to detect a large percentage of patients eventually diagnosed with HIV [2]. In 2006, the CDC guidelines recommended universal screening for persons aged 13–64 [3]. In 2013, the United States Preventive Services Task Force (USPSTF) released an update to their screening guidelines for prevention of HIV infection, endorsing screening all patients aged 15–65 years regardless of perceived risk [3]. One of the concerns with adopting routine HIV screening in our setting has been the popular perception of an unfavorable cost-benefit profile in areas outside large urban cities where HIV prevalence is lower, such as our Midwestern community. However, the CDC has recommended that routine screening be implemented in places where the prevalence of HIV has been noted to ≥0.1 %, and even in places where the prevalence of HIV is not known, the CDC recommends routine screening be implemented unless the prevalence is subsequently determined to be <0.1 % [4].

### History and barriers of HIV screening efforts

Barriers to universal HIV screening can be patient-related or provider-related. Despite the advances in HIV diagnosis and management resulting in decreased mortality from HIV, there may still be a perceived stigma associated with an HIV diagnosis. The World Health Organization has underscored the need for efforts worldwide to reduce stigma and discrimination associated with HIV testing and diagnosis [5, 6]. A focus group survey of 47 US inner-city individuals revealed that fear can be an important patient-driven factor in the avoidance of HIV screening – this may include fear of dying, retaliation against their partners (who are perceived to have infected them), fear of rejection and discrimination and concerns of lack of anonymity [7]. Additionally, there were many misconceptions regarding the epidemiology and transmission of HIV, including fear of becoming infected with HIV while being tested, and poor understanding of the sensitivity of the HIV screening test [7]. Barriers to universal HIV screening is a global issue and these fears are not limited to the United States. A South African study found similar perceptions of stigma associated with HIV, including that HIV-infected individuals deserved their diagnosis as punishment for their offenses, and that people would not befriend them if they were diagnosed with HIV. [8] Cost of testing and inconvenience of testing centers and return visits for counselling may also play roles as patient-driven barriers to HIV screening [7]. Patients may feel invincible and that their self-perceived risk of HIV acquisition is low, therefore do not feel the need to be tested. This has been described by African American men [9] and seems to be on the rise in older adults over age 50 [10]. Finally, patients can avoid testing because they would rather not know their diagnosis than have to disclose it to their partners or families [7], However, patients were more likely to consider HIV screening if the tests were rapid, non-invasive, anonymous and inexpensive [7].

Although the CDC issued recommendations in 2006, a separate World Health Organization document published in 2007 endorsed “Provider-Initiated HIV Testing and Counseling” in healthcare facilities *only* for patients whose presenting symptoms may be consistent with underlying HIV infection (in low-level epidemic situations) [11]. It is not surprising therefore, that it may be difficult for providers to transition from symptom or risk-based screening to universal screening, especially in low-prevalence areas, and there has not been a significant increase in universal HIV screening practices in the United States after those recommendations [12]. Most provider HIV screening practices may differ from their beliefs if they perceive their community to have a higher or lower prevalence of HIV than actually exists [13, 14]. Unfamiliarity with current HIV screening recommendations may be a barrier to adequate screening [15]. Additional barriers to screening may include uncertainty about consent recommendations, concern about patient perception, or patient refusal [16].

An important tool for improving universal HIV screening worldwide is community-based HIV testing and counseling [17]. This includes door-to-door testing and mobile testing units for high-risk groups. These approaches have had higher success rates of earlier HIV diagnosis and linkage to care than provider-based screening; however, the rates of new HIV infections identified with the community-based approach were lower [17]. This community-based approach was successful in many middle-low income countries, but fewer studies have been performed in North America using this approach. Nevertheless, many cost-benefit analyses of universal HIV screening have identified appreciable cost-diagnosis benefits compared to targeted screening, even in adults over age 50 and in lower-risk regions where the prevalence of HIV is >0.1 % [18–23]. Nevertheless, the role of provider-initiated screening of asymptomatic patients for HIV in the primary care setting as part of routine preventive services remains underappreciated.

Emergency departments represent a healthcare “safety net”, and as such are pivotal in the frontlines of HIV screening, diagnosis and linkage to care. In a Midwestern emergency department, more patients were tested with universal screening; this approach identified more new diagnoses, however an opt-in consent approach was used rather than opt-out as recommended by the CDC [24]. Other hospitals and emergency departments have successfully integrated opt-out universal HIV screening into their workflows [25–27]. One Irish hospital emergency department utilized an opt-out triple bloodborne pathogen screen for HIV, hepatitis B and hepatitis C, finding a much higher prevalence of HIV positive individuals using this method than previous estimates of prevalence in the community [28]. In California, a randomized clinical trial comparing the language used in offering HIV screening reported that opt-out language significantly increased the uptake of HIV screening in the emergency department [29].

### Population-based informatics in preventive services

Over the last two decades, the revolution of information technology has transformed healthcare and is now the epicenter of the movement towards an information-driven, patient-centered system [30, 31]. Clinical decision support (CDS) tools successfully enhance chronic disease management, medication management and diagnosis [32]. CDS tools support increased patient involvement in medical decision-making and improved provider-patient relationship [33]. CDS tools have been used to inform healthcare providers of drug-drug interactions and use pharmacogenetics to identify patients at high risk for adverse reactions [34, 35]. Additionally, population-based informatics systems have been implemented to improve preventive services, including mammography and osteoporosis [36, 37]. Recently, similar informatics strategies have been employed for HIV screening in cities with high HIV prevalence such as New York City and New Orleans, documenting significant improvements with electronic health record (EHR) interventions [38]. Innovative strategies for implementation of universal HIV screening are needed to augment provider-initiated and community-based screening efforts.

We designed a quality improvement project to address the need to improve universal HIV screening rates. We hypothesized that the addition of an HIV screening prompt to a CDS would result in improved HIV screening rates within a perceived “low-risk” Midwestern primary care internal medicine practice. We also examined barriers to HIV screening that make the informatics approach to HIV screening different from the use of informatics in other screening programs.

## Methods

### Setting/context

Our quality improvement study took place in the Division of Primary Care Internal Medicine (PCIM), Mayo Clinic in Rochester, Minnesota, located in Olmsted County. The population was 37,453 empanelled patients residing in Rochester and the surrounding rural community. Patients aged 18 to 34, 35 to 49, and 50 to 64 accounted for 20 %, 21 %, and 28 % respectively of the population. Females accounted for 56 % of the patients and 29 % were Mayo Clinic employees. The healthcare providers were 43 internists, 9 nurse practitioners/physician assistants, and 96 internal medicine residents. In 2014, there were 142 people living with HIV in Olmsted County (excluding federal prisoners) [39].

### Root cause analysis

**Fig. 1:**
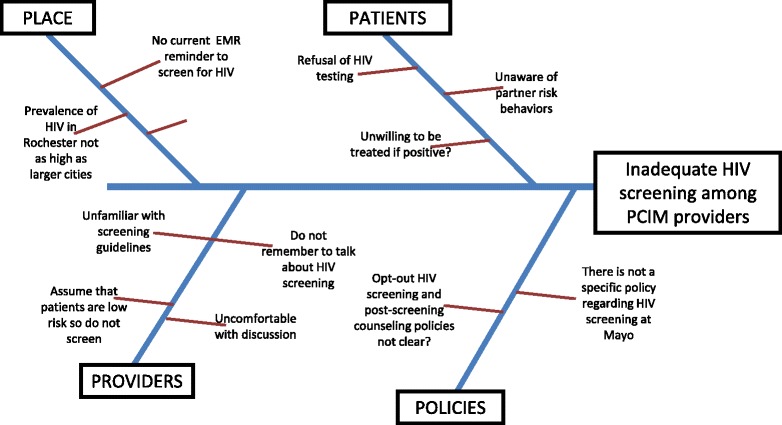
Fishbone diagram depicting root cause analysis of the problem of inadequate HIV screening among PCIM providers

We surveyed 148 PCIM providers prior to the intervention as a means of obtaining a stakeholder analysis and systems audit of the problem of inadequate HIV screening. The REDCap (Research Electronic Data Capture) application was used to create a secure web-based survey which was distributed to PCIM providers via email [40]. The survey assessed providers’ knowledge of current guidelines, behaviors and attitudes towards universal HIV screening, and perceived barriers to successful implementation of universal HIV screening (Additional file 1: eAppendix 1 & 2). Root cause analysis is shown in Fig. 1. Providers received another secure web-based REDCap survey after the intervention to assess the impact of the intervention. Providers who were third year Internal Medicine Residents at the time of the pre-intervention survey and had since graduated were not available to respond to the post-intervention survey. Similarly, providers who were first year Internal Medicine Residents at the time of the post-intervention survey could not have responded to the pre-intervention survey, as they were not residents in our clinic at that time.

### Clinical decision support development

**Fig. 2:**
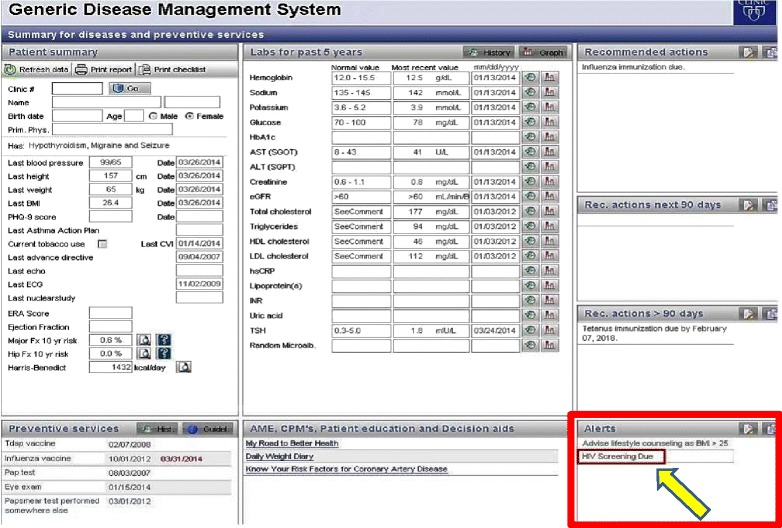
Generic Disease Management System (GDMS) interface: “HIV Screening Due” appears under the Alerts for eligible patients

HIV screening alert notifications for the providers were developed for this study from the Generic Disease Management System (GDMS), a web-based knowledge delivery solution integrated with the Mayo Clinic’s EHR [41]. GDMS uses clinical data in the EHR to determine what preventive services or chronic disease management tasks are needed, calculates when they are due, and alerts the provider. Logic-based rules, based on published clinical guidelines and validated for other clinical conditions, are used to determine the recommended actions for each patient [42]. GDMS delivers these rule-based alerts via a proprietary viewer interface called Synthesis, shown in Fig. 2. Using the viewer, the alerts fit into the workflow of the patient visit, which is a crucial part of a successful CDS system [43]. Providers also receive a printout of the GDMS output to enable provider-patient engagement and discussion of the recommendations during the appointment. GDMS provides both alerts and recommended actions; alerts are viewable to providers on screen only, while recommended actions are viewable on screen and on the printout. For the initial intervention pilot, the HIV screening recommendation was programmed as an alert only (i.e. viewable only electronically through GDMS in the EHR).

Logic was used to prompt universal HIV screening for patients between the ages of 18 and 65 who have never been screened for or diagnosed with HIV. Although guidelines recommend screening patients between ages 13–17, these patients were not in the adult study population and were not included. A patient was considered to have been previously screened for HIV if the patient had any of the screening tests shown in Additional file 1: eTable 1. GDMS also searched the patient’s problem list for HIV diagnosis codes listed in Additional file 1: eTable 2 as exclusion criteria. If these criteria were met after real-time assessment by the web services, the HIV rule was applied and GDMS alerted the provider “HIV Screening Due” (Fig. 2). Of note, if the alert was delivered and the patient indicated to the provider during their visit that they have received an HIV screen elsewhere; the provider can note this using a disposition function stored in the EHR so that an alert is not generated in subsequent visits.

### Clinical decision support implementation

Medical records of patients seen in the PCIM practice from January 1, 2014 to April 30, 2014 were queried to identify the baseline HIV screening rate. We met with the providers to share the data on current screening practices and provide education about current screening recommendations. Finally, we showed providers the details of the intervention, demonstrating exactly what the GDMS screen would look like with the intervention in place (Fig. 2). These meetings were followed by several educational and reminder emails with similar information. After a run in period where rules were tested and corrected, final implementation was audited with a sample of 10 patients and showed 100 % accuracy of HIV screening recommendations.

### Outcome measures

The numbers of HIV screening tests performed by utilizing the HIV screening codes described in Additional file 1:eTable 1 and excluding those with diagnosis codes described in Additional file 1: eTable 2.

**Table 1:** Baseline characteristics

	Pre-intervention (*N* = 6070)	Post-intervention (*N* = 6526)	*p* value
Patients screened, (%)	109 (1.80)	218 (3.34)	<0.001
Mean age [SD], yrs.	48.9 [12.3]	48.4 [12.6]	0.01
Female sex (%)	3464 (57.1)	3535 (54.2)	0.001
White race, (%)	5105 (84.1)	5533 (84.8)	0.21
SD standard deviation			

### Data collection

Pre-intervention and post-intervention screening data were obtained from 1/1/14-4/30/14 and 5/1/14-12/31/14, respectively. Patients with a previous diagnosis of HIV or HIV-related disorder were excluded.

### Statistical analysis

JMP software version 10.0 (Windows ©2005 SAS Institute, Inc) was used for analysis of the HIV screening data and the provider survey results. We analyzed patient characteristics before and after the intervention using the chi-square test for categorical variables and the t-test for continuous variables. Logistic regression was used for modeling with odds ratios and 95 % confidence intervals (CI) reported; the likelihood ratio test statistic was used for testing of significance.

## Results

Six thousand seventy and six thousand five hundred twenty-six patients were seen prior to and after the GDMS alert intervention respectively. The cohort consisted of a predominantly white and middle-aged population (Table 1). 327 patients were screened for HIV in 2014. 109 (1.80 %) eligible patients were screened prior to the GDMS alert intervention; 218 (3.34 %) eligible patients were screened after the intervention (difference of −1.54 % [−2.1 %, −0.99 %], *p* < 0.001). As a cohort, there were no age or sex differences in screening before and after the intervention, with a mean age of 48 years in both groups (Table 1). Prior to the intervention, African Americans were more likely to have been screened for HIV (OR 3.86 (2.22, 6.71; *p* < 0.001) than Whites, but this effect decreased significantly after the intervention (OR 1.90 (1.12, 3.21; *p* = 0.03) (Table 2). No new HIV diagnoses or false positives were identified.

**Table 2:** Odds Ratios and 95 % Confidence Intervals for HIV screening within demographic groups before and after GDMS intervention

Demographic variable:		Pre-intervention	*p* value^a^	Post-intervention	*p* value^a^
		Odds Ratio (95 % CI)		Odds Ratio (95 % CI)	
Age	18–24	0.34 (0.11, 1.11)	0.037^b^	1.29 (0.75, 2.20)	0.37 ^b^
	25–39	1	–	1	–
	40–49	0.35 (0.20, 0.63)	<0.001^b^	0.50 (0.33, 0.78)	0.001^b^
	50+	0.27 (0.18, 0.42)	<0.001^b^	0.57 (0.42, 0.79)	<0.001^b^
Sex	Female	1	–	1	–
	Male	1.26 (0.86, 1.85)	0.23	1.21 (0.92, 1.59)	0.1639
Race	White	1	–	1	–
	Asian	0.88 (0.32, 2.42)	0.80^c^	1.71 (1.02, 2.85)	0.06^c^
	AA	3.86 (2.22, 6.71)	<0.001^c^	1.90 (1.12, 3.21)	0.03^c^
	Other^d^	2.48 (1.27, 4.86)	0.02^c^	1.41 (0.76, 2.63)	0.30^c^
	Unknown	3.44 (1.23, 9.64)	0.05^c^	2.25 (1.03, 4.91)	0.07c

*AA* African American, *CI* confidence interval

^a^Likelihood ratio test

^b^Compared to subjects aged 25–39

^c^Compared to white race

^d^Includes Native Hawaiians, Pacific Islanders, Native Americans

### Survey results

**Fig. 3:**
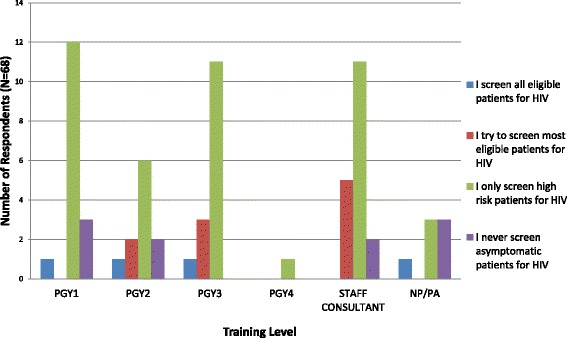
Chart showing pre-intervention survey response regarding provider HIV screening practice, by training level

Of the 148 Internal Medicine providers surveyed before the intervention, 68 providers (45.9 %) responded. 63 % of responding providers did not universally screen all eligible patients for HIV, most commonly citing that they did not remember to discuss screening (52 %). 33 % were not familiar with current screening guidelines, and 25 % of providers did not believe in screening patients they perceived to be “low-risk”. 4 % of providers indicated that they were uncomfortable with the discussion. Figure 3 describes the survey responses regarding screening practices, by provider training level. Across all training levels, before the intervention, the overwhelming practice was to screen only perceived “high risk” patients for HIV. However, prior to the intervention, 88 % of the responding providers indicated that they would screen more patients for HIV if the GDMS system had an HIV screening prompt.

**Fig. 4:**
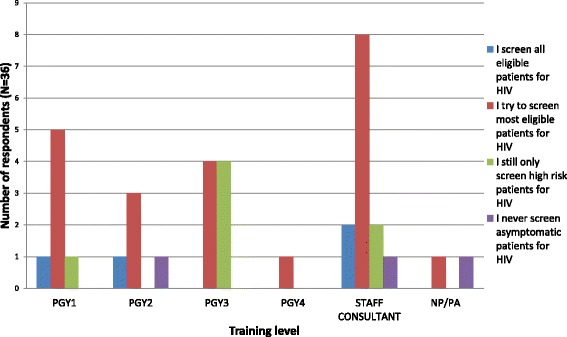
Chart showing post-intervention survey response regarding provider HIV screening practice, by training level

After the intervention, the providers were re-surveyed and 36 (24.3 %) responded. 78 % of responding providers were aware of the GDMS electronic prompt. Ten providers (27 %) indicated that they still only screened high-risk patients for HIV; 75 % indicated that they would be more likely to screen if the recommendation was viewable to patients also. 64 % of responding providers felt that the intervention helped to educate them about HIV screening, and 42 % felt that more of their patients agreed to the test after the intervention was implemented. Figure 4 describes the survey responses regarding screening practices, by provider training level. Across all training levels, after the intervention, more providers endorsed that they tried to screen most eligible patients for HIV.

## Discussion

This CDS intervention improved the HIV screening practices of primary care providers. However, the small absolute numbers may reflect the continued perception that our community population is at low risk for HIV infection. In an Ohio community Internal Medicine training program, HIV screening rates were successfully increased from 0.4 % to 16.9 % [44]. In that program, a trained medical assistant suggested HIV screening at the beginning of the visit. However, this study differs from ours in that (1) their clinic serves a largely uninsured, medically indigent or low income population, (2) the clinic served fewer patients than our clinic and (3) they offered HIV testing to all patients entering the clinic regardless of prior HIV screening status [44].

African Americans continue to be disproportionately affected by the HIV epidemic, with 44 % of new HIV infections occurring in this group [1]. Perhaps for this reason, African Americans in this cohort were screened more often than other racial/ethnic groups (Table 2). Of interest, while the proportion of African Americans screened before and after the intervention remained constant, the proportion of White patients screened after the intervention significantly increased from 1.47 % to 3.02 %, OR 2.09 (1.59, 2.75), *p* < 0.001 (Table 3). Additionally, the age groups that seemed to benefit the most from the intervention were the youngest (18–24) and oldest (50+) cohorts – both of these groups showed significant improvement in overall screening rates after the intervention. Provider initiated screening in these age groups is especially important. Youth in the 18–24 year old age group tend to feel invincible and do not perceive themselves at risk for HIV, and older adults over age 50 tend to consider other disorders of aging to explain symptoms before thinking of risk for HIV [1]. Our results indicate that an electronic reminder can facilitate a more truly universal screening program, with all gender, race/ethnic groups and age groups being screened according to eligibility.

**Table 3:** Comparison of overall HIV screening rates and by demographic group before and after GDMS intervention

Demographic Category	Pre intervention HIV screening (%) *n* = 6070 eligible	Post Intervention HIV screening (%); *n* = 6526 eligible	Odds Ratio (95 % CI)	*p* value^a^
All eligible	1.80	3.34	1.89 (1.50, 2.38)	<0.001
Sex Female	1.62	3.06	1.92 (1.38, 2.66)	<0.001
Sex Male	2.03	3.68	1.84 (1.32, 2.56)	<0.001
Age 18–24	1.42	6.14	4.54 (1.32, 15.6)	0.005
Age 25–39	4.03	4.85	1.21 (0.83, 1.76)	0.31
Age 40–49	1.47	2.51	1.73 (0.94, 3.18)	0.07
Age 50+	1.13	2.83	2.54 (1.76, 3.68)	<0.001
Race: White	1.47	3.02	2.09 (1.59, 2.75)	<0.001
Race: AA	5.44	5.57	1.03 (0.51, 2.09)	0.94
Race: Asian	1.29	5.04	4.05 (1.35, 12.2)	0.005
Race: Other^b^	3.57	4.20	1.18 (0.49, 2.83)	0.71
Race: Unknown	4.88	6.54	1.37 (0.39, 4.83)	0.63

*AA* African American, *CI* confidence interval

^a^Likelihood ratio test

^b^Includes Native Hawaiians, Pacific Islanders, Native Americans

Despite updates to HIV screening recommendations, universal screening rates remain unsatisfactory. In a 2009 study of Internal Medicine providers, 88 % reported knowledge of universal screening recommendations, yet only 50 % endorsed any increase in their screening rates [13]. Perceived conflict with other patient priorities, anticipated patient refusal, or concerns about reimbursement often serve as barriers to optimal screening [13]. Therefore, risk-based screening remains popular despite widespread knowledge about the benefits of universal screening [12].

It is easy to envision that screening practices can be improved with education and electronic screening tools in larger urbanized areas such as the New York Metropolitan area or New Orleans [38]. In these cities, the “at risk” population is considered because of behavioral differences and larger population size. In smaller cities, universal screening strategies may be more difficult to implement since providers’ perceptions of screening need are less obvious. Some of our providers believed that the community served by our institution is over-served and our focus should be on patients living in rural areas. Despite geographic differences in access to care, the premise of universal HIV screening is that risky behaviors leading to acquisition of HIV are present across all social, ethnic, economical and age strata.

The CDS itself may pose certain barriers. Healthcare providers may not be aware that it exists or know how to use the information within the CDS [45]. Additionally, they may encounter conflict when utilizing the CDS to achieve certain patient-care goals when these goals are not shared by the patients [45]. Most patients do not come to the doctor’s office thinking about HIV screening. Furthermore, there are many barriers to HIV screening. First, many healthcare providers may not be aware of the 2006 CDC recommendations for universal HIV screening. In the past, providers needed separate written consent and pretest and posttest counseling to screen for HIV, and some providers do not realize these actions are no longer required in most states, with few exceptions [4]. Because of such education gaps, a study like this often requires a multi-centered approach with both electronic reminders and provider education. While such an approach may lead to overall improvement in screening, it makes it difficult to determine if the effect is due to the alert or the education, or both; this is a common issue with positive CDS studies [46].

Providers also feel insecure about discussing HIV testing with patients, as this may strain the patient-provider relationship. Providers may try to avoid making the patient feel that they are passing judgement on their sexual risk factors, and in doing so may completely avoid discussions about sexually transmitted disease screening in the absence of patient prompting. This alludes to a persistent perceived stigma associated with HIV screening and diagnosis. Many patients doubt the confidentiality of HIV testing, and may deny being at risk for HIV [16]. Because patients need increased reassurance regarding confidentiality, it is perceived that delivery of HIV test results requires an extra layer of security. If patients can be reasonably reassured that HIV screening is as routine as their cancer or cholesterol screening, and that all patient encounters and test results are confidential regardless of type of discussion or test, the provider-patient relationship could likely be maintained without conflict.

### Future enhancements

Information technology regarding HIV screening requires enhancements in other areas. Since 1985, donated blood has been tested for HIV, therefore patients who have had donated blood accepted in the past must be HIV-negative. However, if the blood donor database is not synchronized with the patient’s medical record, then it would appear that the patient was never screened for HIV. A useful enhancement would be to create a background link between the medical record and blood donor database so that providers considering recommending universal HIV screening would be alerted if the patient had recently *successfully* donated blood. This would also provide a counterbalance measure to avoid over-testing in patients whose donated blood recently passed infectious diseases screening tests. The percent increase in HIV screening rate in the PCIM clinic was modest at best, and absolute numbers were still disappointing. While this particular quality improvement project did not identify any newly diagnosed HIV patients, the opportunity still exists to further increase both provider confidence in the necessity of universal screening and improve the ease of recommending this screening to their patients.

Notifying patients through patient online services, or phone calls makes universal HIV screening more convenient because the patients may be screened concurrently with other recommended labs prior to visiting their provider. Utilization of online reminders may not be desirable however, as they preclude patients from being able to ask questions regarding the recommendations, and telephone recommendations would impose a time–cost burden on nurses or desk staff. Provider audits and addition of HIV screening to institution performance metrics may also complement the electronic prompt system [43]. Self-auditing has been shown to improve HIV screening rates in a Midwest internal medicine residency practice [47]. There is a call to action to further enhance the contribution of health information technology to improve the clinical decision-making capacity of providers [48]. Though their direct impact on patient health outcomes (e.g. mortality) still needs to be studied, automated CDS systems increase provider performance with respect to prevention, diagnosis, and chronic disease management, and should not be overlooked when considering areas for practice improvement [32].

### Limitations

The GDMS HIV screening prompt was available only electronically to the providers as an “*alert*” through the EHR, but was not available on the patient printout. The printout serves as a starting point for discussions between patients and providers about recommended preventive services. Most providers indicated their willingness to screen more often if the prompt was a “*recommended action*” so that patients could recognize that this is a part of the normal preventive service recommendations and not an assumption about their sexual habits. This planned upgrade should boost HIV screening in our practice as a routine part of the shared decision-making process at clinical visits. With this further intervention, we hope to eliminate provider insecurity with HIV screening discussions and patient insecurity with perceived judgement or stigma associated with screening.

While it would have been ideal to determine the true number of opportunities to intervene and screen for HIV, including non-face-to-face encounters, this was not feasible, therefore the surrogate for this was the number of appointments. There were multiple unique appointments per patient, and it was difficult to determine exactly which appointment resulted in the screening opportunity, as patients do not always perform tests immediately after an appointment, and sometimes HIV screening could have been batched with “next visit” future testing. For the same reason, it was not feasible to obtain reliable data on number of HIV tests *ordered* vs. those *completed*, as tests often have to be re-ordered when patients miss lab appointments. Similarly, it was not feasible to identify which patients opted-out of HIV screening despite provider recommendation, as this was not mentioned in medical notes. In addition, records from the Family Medicine and outreach clinic practices were excluded; this could have introduced a selection bias as screening practices may be different in other local primary care practices.

Furthermore, while it would have been interesting to evaluate screening tests by provider training level, it was not possible to do so accurately, as our electronic program only maps current providers to appointments. Since a majority of third year medical residents had graduated by the time the data was collected, their names could no longer be mapped to the appointments they had prior to graduation. Finally, applicability of these quality improvement practices outside our institution could potentially be limited with the use of GDMS only at this institution, but the premise of our study with an electronic prompt and CDS can be applied to any electronic medical record.

## Conclusions

Universal HIV screening can be significantly increased with the use of electronic CDS alerts at the point of care. This HIV screening enhancement has been sustainable and continues to be a part of the CDS system in our institution. Given the low absolute screening rates, there is still substantial room for improvement in universal HIV screening, especially among perceived “low prevalence areas.” These results can serve as a benchmark to other institutions and a starting point for further development of ways to increase HIV screening.

## Abbreviations

CDC, Centers for Disease Control and Prevention; CDS, clinical decision support; EHR, electronic health record; GDMS, generic disease management system; HIV, human immunodeficiency virus; PCIM, primary care internal medicine; REDCap, research electronic data capture; USPSTF, United States Preventive Services Task Force

## Additional file

Additional file 1: Appendix Items. eTable 1. Labs used to check for “Prior HIV Screen”. eTable 2. Codes used to check for HIV diagnosis. eAppendix 1: Pre-intervention Survey: HIV Screening in PCIM (Primary Care Internal Medicine). eAppendix2: Post-intervention Survey: HIV Screening in PCIM (Primary Care Internal Medicine). (PDF 250 kb)
