# Supplementary material for: Assessment and improvement of HIV screening rates in a Midwest primary care practice using an electronic clinical decision support system: a quality improvement study
Source: BMC Med Inform Decis Mak. 2016 Jul 4;16:76. doi: 10.1186/s12911-016-0320-5 (PMC4932674; doi:10.1186/s12911-016-0320-5)
Supplement: Additional file 1: — Appendix Items. eTable 1. Labs used to check for “Prior HIV Screen”. eTable 2. Codes used to check for HIV diagnosis. eAppendix 1: Pre-intervention Survey: HIV Screening in PCIM (Primary Care Internal Medicine). eAppendix2: Post-intervention Survey: HIV Screening in PCIM (Primary Care Internal Medicine). (PDF 250 kb) [file 12911_2016_320_MOESM1_ESM.pdf]

APPENDIX
Assessment and improvement of HIV screening rates in a Midwest primary care practice using an electronic clinical decision support system (*Marcelin, JR et al*.)

eTable 1. Labs used to check for "Prior HIV Screen"

ROCLIS/ROCMIC Institution Test Code	Test Description
26312-ROCLIS	HIV-1/-2 Ab Screen, S
81762- ROCLIS	HIV-1/HIV-2 Ab Rapid, P
9333-ROCLIS	HIV-1/-2 Ab Evaluation, S
32471-ROCLIS	HIV-1/HIV-2 Ab Rapid, P
M62420-ROCLIS	HIV-1/-2 Ag and Ab Screen, S-PHDICT

eTable 2. Codes used to check for HIV diagnosis

Description	IDC9	Code
HIV	042	2200670
HIV Asymptomatic	V08	2214827
HIV Encephalopathy	042	2216035
HIV Nephropathy	042	2216955
HIV or AIDS In Preg Antepartum	647.63	2222274
HIV or AIDS In Preg Postpartum	647.64	2222275
HIV Positive	042	2216000
HIV Serology Nonspecific	795.71	2211651
HIV Type2 Infection NOS	079.53	2200949
HIV w/ AIDS	042	2216001
Dementia from HIV	042	2219448
Dementia from HIV w/ Behav. Problem	042	2219449
Facial Ids w/ HIV (Facial Lipodystrophy Syndrome w/ HIV)	272.6	2224370
Facial Lipodystrophy Syndrome w/ HIV	272.6	2224370
Wasting Syndrome from HIV	799.4	2216995

APPENDIX
Assessment and improvement of HIV screening rates in a Midwest primary care practice using an electronic clinical decision support system (*Marcelin, JR et al*.)

eAppendix 1:
Pre-intervention Survey: HIV Screening in PCIM (Primary Care Internal Medicine)

1. Do you screen all eligible patients for HIV?
   A. Yes
   B. No
   C. I'm not sure which patients are eligible for screening

2. Which of the following best describes your HIV screening practices?
   A. I screen all patients for HIV according to current guidelines
   B. I screen most patients for HIV
   C. I screen only high risk patients for HIV
   D. I never screen asymptomatic patients for HIV

3. If you have not screened patients for HIV, what factor(s) lead to this decision?
   A. Not familiar with current screening guidelines
   B. Don't remember to discuss screening with patients
   C. Patients refuse HIV screening
   D. Unaware if patients have been screened before
   E. Don't believe in screening patients if I perceive them to be lower risk
   F. This discussion is too uncomfortable or awkward
   G. Unsure if consent is required
   H. Other (please specify)

4. Are you familiar with current HIV screening guidelines?
   A. I am familiar with the guidelines
   B. I am aware they exist but do not know exactly what they are
   C. I did not know that there were specific HIV screening guidelines

5. If GDMS had an HIV screening prompt, would you screen more patients for HIV?
   A. Yes
   B. No
   C. Not applicable; I already screen every eligible patient according to guidelines

6. What is your gender?
   A. Female
   B. Male

7. What is your current post-graduate year?
   A. PGY-1
   B. PGY-2
   C. PGY-3
   D. PGY-4 or higher
   E. Staff Physician
   F. NP/PA or other non-physician provider

8. Do you have comments or suggestions on what would help you to screen more patients for HIV?

APPENDIX
Assessment and improvement of HIV screening rates in a Midwest primary care practice using an electronic clinical decision support system (*Marcelin, JR et al*.)

eAppendix2:
**Post-intervention Survey: HIV Screening in PCIM (Primary Care Internal Medicine)*

1. Are you aware of the HIV screening recommendation prompt on GDMS?
   A. Yes
   B. No

2. Since the implementation of the HIV screening prompt on GDMS, which of the following best describes your HIV screening practices?
   A. I now screen all eligible patients for HIV according to current guidelines
   B. I try to screen most eligible patients for HIV
   C. I still screen only high risk patients for HIV
   D. I never screen asymptomatic patients for HIV

3. If you have utilized the HIV screening prompt on GDMS, what has been the patient response?
   A. More patients agree to the test
   B. Patients still refuse the test
   C. It has not made a difference

4. Would you be more likely to screen for HIV if GDMS made the recommendation available for the patient also?
   A. Yes
   B. No
   C. Not Necessarily

5. Did the intervention help to educate you about HIV screening?
   A. Yes
   B. No

6. Do you think providers need more education about HIV screening?
   A. Yes
   B. No

7. Do you have enough time to discuss HIV testing at the point of care?
   A. Yes
   B. No

8. Should HIV testing discussion be initiated by rooming personnel prior to the face-to-face provider visits?
   A. Yes
   B. No

9. What is your gender?
   A. Male
   B. Female
   C. Other
      - Define Other ______________________________________

10. What is your current post-graduate year?
   A. PGY-1
   B. PGY-2
   C. PGY-3
   D. PGY-4 or higher (but still in training)
   E. Staff Physician
   F. NP/PA or other non-physician provider

11. Do you have any last comments that you believe would be helpful in improving HIV screening in PCIM?
